# Resilience acts as a moderator in the relationship between infertility-related stress and fertility quality of life among women with infertility: a cross-sectional study

**DOI:** 10.1186/s12955-019-1099-8

**Published:** 2019-02-15

**Authors:** Yifei Li, Xin Zhang, Meng Shi, Shuaishuai Guo, Lie Wang

**Affiliations:** 1 Department of Social Medicine, School of Public Health, China Medical University, No.77 Puhe Road, Shenyang North New Area, Shenyang, 110122 China; 2 English Department, School of Fundamental Sciences, China Medical University, No.77 Puhe Road, Shenyang North New Area, Shenyang, 110122 China; 3 Reproductive Medicine Center, Shenyang Women’s and Children’s Hospital, No.87 Danan Road, Shenhe Area, Shenyang, 110000 China

**Keywords:** Infertility, Quality of life, Infertility-related stress, Resilience, Moderating role

## Abstract

**Background:**

Infertility-related stress can seriously impact the fertility quality of life (QoL) of infertile women. Resilience, as a coping resource, can effectively combat psychological stress. This study aimed to evaluate the fertility QoL of infertile women and to examine whether resilience moderates the association of infertility-related stress with fertility QoL.

**Methods:**

This cross-sectional study was conducted in northeast of China from December 2017 to February 2018. Out of 559 women outpatients with infertility, 498 (89.1%) completed self-reported questionnaires including the FertiQoL Scale, Fertility Problem Inventory (FPI) and Connor-Davidson Resilience Scale (CD-RISC). Hierarchical multiple regression analysis and simple slope analysis were applied to explore the influencing factors related to fertility QoL as well as to examine the moderating effect of resilience on the association of infertility-related stress with fertility QoL.

**Results:**

The mean FertiQoL score was 64.54 ± 16.90 among the participants. Household monthly income and causes of infertility were significantly related to fertility QoL. In addition, infertility-related stress was negatively related to fertility QoL, and resilience was positively associated with fertility QoL, explaining 36.3% of the variance. Resilience moderated the association of infertility-related stress with fertility QoL. Specifically, the effect of infertility-related stress on fertility QoL varied by low(1 SD below the mean, *B* = - 0.496, *β* = - 0.714, *P* < 0.001), mean (*B* = - 0.293, *β* = - 0.422, *P* < 0.001) and high (1 SD above the mean, *B* = - 0.090, *β* = - 0.130, *P* < 0.001) levels of resilience. The higher resilience was, the weaker the effect of infertility-related stress on fertility QoL was.

**Conclusions:**

Overall, women with infertility in China had relatively low FertiQoL scores. Resilience influenced the association of infertility-related stress with fertility QoL. Infertile patients’ psychological status must be addressed and adequate resilience-based interventions such as mindfulness-based skills should be provided to improve fertility QoL of women with infertility.

## Background

According to Zegers-Hochschild et al., the latest definition of infertility is “a disease characterized by the failure to establish a clinical pregnancy after 12 months of regular, unprotected sexual intercourse or due to an impairment of a person’s capacity to reproduce either as an individual or with his/her partner” [1]. Worldwide, infertility is a prevalent condition among women aged 15 to 49 years. Previous studies have reported that 15.5% of reproductive-age women in the United States suffer from infertility [2], as well as 24% in France [3] and 25% in China [4]. Infertility can lead to various physical, psychological and societal consequences, such as depression, anxiety, stigma and social isolation, which may significantly impact patients’ fertility quality of life (QoL) [5–7].

QoL is defined by the World Health Organization (WHO) as individuals’ perceptions of their position in life in the context of culture and the value systems where they live [8]. Accordingly, fertility QoL involves a reflection of infertile patients’ life status during their infertile period in a broad sense. A large number of studies revealed that compared with the fertile counterparts, infertile women experienced poorer QoL during the period of infertility [9–11]. In addition, poor fertility QoL of infertile women was shown to be negatively related to treatment compliance and could cause latent economic burdens on their families and communities [12]. However, the identification of the influencing factors of fertility QoL makes it possible to conduct targeted interventions and care activities in an integrated way to improve the fertility QoL of women with infertility, which necessitates our study.

The impact of infertility-related stress on fertility QoL is an increasingly important topic in recent scientific research. Infertility-related stress refers to perceived stresses from one’s social networks, marital relationships, and physical and mental health due to infertility [13]. In many societies, especially in China, infertility and the consequent childlessness are often correlated with stigma and guilt [6, 14]. As a result, infertile women may have a strong sense of loneliness and social stress. In addition, Ganth et al. found that most infertile couples were dissatisfied with their marital lives, which could predispose women to additional marital stress [15]. Furthermore, it is well accepted that both a diagnosis and infertility treatment can induce heavy physical and psychological stress [16]. Several well-documented studies have indicated that infertile women seem to carry a much heavier burden and stress than their male counterparts [17, 18]. Therefore, women with infertility may perceive considerable infertility-related stress that can result in a life crisis and significantly impact their fertility QoL.

As a positive psychological resource, resilience has attached increasing importance in clinical practice. Resilience is defined as the developable capabilities to rebound or bounce back from tragedy, frustration and failure or even positive events [19]. Resilient patients are usually considered to possess self-esteem, believing in one’s own self-efficacy and having effective problem-solving skills to cope with stress [20, 21]. Many previous studies have demonstrated that resilience has direct and positive effects on patients’ QoL [22, 23]. Specifically, resilience was found to be strongly and positively related to QoL among women with infertility [24]. In addition, prior studies have supported the mediating effect of resilience resources on QoL [22, 25]. Resilience can also act as a moderator to buffer the effect of antecedent indicators on QoL. For example, Palm-Fischbacher et al. reported that resilience could act as a moderator in the association of chronic stress with physical health among young women [26]. Another study by Rainone et al. showed that resilience could moderate the relationship between affective disorders and QoL among patients with multiple sclerosis [27]. Since the protective effect of resilience on QoL and its negative effect on perceived stress have been widely reported [22–28], resilience seems to be able to moderate the association of infertility-related stress with fertility QoL. In other words, the effect of infertility-related stress on the fertility QoL can be affected by the levels of resilience patients possess.

However, to the best of our knowledge, extant studies have not yet explored resilience as a moderator in the relationship between infertility-related stress and fertility QoL among women with infertility. Therefore, in the present study, we aimed to evaluate the fertility QoL among these patients to examine whether resilience moderates the association of infertility-related stress with fertility QoL and to find solutions to improve fertility QoL.

## Methods

### Ethics statement

The study protocol was in accordance with the ethical standards and was approved by the Ethics Committee of Shenyang Women’s and Children’s Hospital. Written informed consent was obtained from each participant. Information collected from all participants was kept confidential and anonymous.

### Study design and data collection

This cross-sectional study was carried out in outpatients diagnosed with infertility from December 2017 to February 2018. All participants were recruited at the Reproductive Medicine Center of Shenyang Women’s and Children’s Hospital in Liaoning Province, northeastern China. The inclusion criteria were as follows: 1) women outpatients diagnosed with infertility and aged over 18 years; 2) women who received in vitro fertilization and embryo transfer (IVF-ET) treatment; 3) women who were literate and could communicate well in Chinese; and 4) women who were willing to voluntarily complete a multi-item questionnaire. The exclusion criteria included the following: 1) women who had other major diseases (such as cancers and severe cardiovascular and cerebrovascular diseases) at the present stage; 2) women who had a psychiatric history; and 3) women who had intellectual and/or cognitive impairments. After obtaining their written informed consent for this study, a self-reported questionnaire was distributed to each eligible participant, and clinical data were collected from their medical records. Questionnaires with any missing data were excluded from statistical analyses. Finally, out of the 559 eligible participants, 27 patients declined to participate and 34 questionnaires were excluded. In total, 498 complete responses were received (89.1%) in the present study.

### Measures

#### Measurement of fertility QoL

We used the Chinese version of the FertiQoL Scale to measure fertility QoL in infertile women [29]. The Chinese version of the FertiQoL Scale has been translated from the international FertiQoL questionnaire and consists of two modules: a core FertiQoL module and an optional treatment module [30]. The optional FertiQoL treatment module was not used in this study. The Chinese version of the core FertiQoL module contained two general items and 22 specific items covering mind-body, relational, social and emotional domains (e.g., Do your fertility problems interfere with your day-to-day work or obligations?). Each item was rated on a five-point Likert scale from 0 to 4. The total raw scores were computed and transformed to standard scores ranging from 0 to 100, with a higher score reflecting a higher fertility QoL. The Chinese version of the FertiQoL scale has been widely used and has shown good reliability and validity among Chinese populations [29, 31]. In present study, the Cronbach’s alpha coefficient of the FertiQoL Scale was 0.925.

#### Measurement of infertility-related stress

The Fertility Problem Inventory (FPI) compiled by Newton et al. is a useful tool to measure the level of infertility-related stress in five dimensions, namely, social concern, relationship concern, sexual concern, the need for parenthood, and rejection of a child-free lifestyle [32]. In this study, the infertility-related stress of the participants was measured with the Chinese version of FPI, which comprises 46 items (e.g., I can’t help comparing myself with friends who have children) [33]. Each item was scored using a six-point Likert scale ranging from 1 (do not agree) to 6 (totally agree). The overall scores ranged from 46 to 276, and a higher score indicated higher perceived fertility-related stress. The Chinese version of the FPI was reported to have adequate reliability and validity [33]. The Cronbach’s alpha coefficient of FPI was 0.823 in this study.

#### Measurement of resilience

The Chinese version of the widely used Connor-Davidson Resilience Scale (CD-RISC), developed by Connor et al., was used to assess resilience [34, 35]. The Chinese version of CD-RISC is a 25-item scale (e.g., coping with stress strengthens me) and the items are scored on a five-point scale ranging from 0 (not true at all) to 4 (true nearly all the time). A sum score was calculated with higher total scores reflecting higher levels of resilience. The Chinese version of CD-RISC has shown adequate reliability and validity [35, 36]. The Cronbach’s alpha coefficient of the Chinese version of CD-RISC was 0.943 in this study.

#### Demographic characteristics

Demographic characteristics included age, educational background, residence and household monthly income. Educational background was categorized into senior high school or below, junior college and college or above. Residence was classified into rural and urban areas. Household monthly income was divided into ≤4000 and > 4000 yuan (approximately 600 dollars).

#### Clinical variables

Four clinical variables were measured including pregnancy history, surgical history, infection history and causes of infertility. Pregnancy history and surgical history were categorized into yes or no answers. Infection history was defined as “yes” if the respondents had suffered from sexually transmitted infections or had a history of induced abortion infection; otherwise, it was categorized as “no”. The causes of infertility were divided into three groups: female factors (e.g., ovulation disorders, endometriosis), mixed factors (female factors mixed with male factors such as oligospermia, erectile dysfunction, etc.) and unexplained reasons.

### Statistical analysis

The mean scores of FertiQoL with different categories of demographic and clinical variables were examined by Student’s *t*-test or one-way ANOVA. Pearson’s correlation analysis was used to analyze the correlation among fertility QoL, infertility-related stress and resilience. Hierarchical multiple regression analysis was applied to investigate the factors in relation to fertility QoL as well as to explore the moderating role of resilience on the association of infertility-related stress with fertility QoL. All variables that were associated with fertility QoL in univariate analysis (*P* < 0.05) were entered into the hierarchical multiple regression model. In the model, age and potential control variables were entered in step 1. Infertility-related stress and resilience were added in step 2. Finally, the product of infertility-related stress and resilience was added in step 3. The hypothesis of the moderating effect of resilience was supported if the interaction was significant, and simple slope analysis was conducted to visualize the interaction term. The variables in the model were centralized before regression analysis was conducted. All the statistical analyses above were performed with SPSS for Windows (version 20.0), with a two-tailed *P*-value of < 0.05 considered to be statistically significant.

## Results

### Description of the participants and fertility QoL

Demographic and clinical characteristics and group differences in fertility QoL are presented in Table 1. The age range of participants varied from 19 to 40 years (mean: 32.19 ± 3.83). The proportion of the participants with an education background level at senior high school or below was 39.6% (197); 77.5% (386) of the participants lived in urban areas. There were 309 (62.0%) participants who had a household monthly income exceeding 4000 yuan. With regard to clinical variables, 45.8% (228) and 41.2% (205) of the participants had a history of pregnancy and surgical history, respectively. Only 7.2% (36) of the participants had an infection history. There were 299 (60.0%), 56 (11.3%) and 31 (6.2%) patients suffering from infertility because of female factors, male factors and mixed factors, respectively. Participants whose monthly income exceeded 4000 yuan experienced a higher level of fertility QoL compared with patients whose household monthly income was below 4000 yuan (*t* = 2.446, *P* = 0.015), and the patients with infertility due to female factors reported lower fertility QoL than the other groups (*t* = 4.079, *P* = 0.007).

**Table 1:** FertiQoL scores by demographic and clinical characteristics

Variables	n	%	FertiQoL			
			Mean	SD	F/*t*	*P*-value
Demographic variables						
Educational background					0.914	0.401
Senior high school or below	197	39.6	65.78	16.70		
Junior college	111	22.3	64.07	16.60		
College or above	190	38.1	63.53	17.29		
Residence					1.275	0.203
Rural area	112	22.5	66.33	16.22		
Urban area	386	77.5	64.02	17.08		
Household monthly income (yuan)					2.446	0.015
≤ 4000	189	38.0	62.19	17.57		
> 4000	309	62.0	65.98	16.34		
Clinical variables						
Pregnancy history					0.467	0.641
Yes	228	45.8	64.16	15.76		
No	270	54.2	64.86	17.83		
Surgical history					1.874	0.062
Yes	205	41.2	62.85	17.57		
No	293	58.5	65.73	16.34		
Infection history					1.737	0.083
Yes	36	7.2	59.84	20.16		
No	462	92.8	64.91	16.59		
Causes of infertility					4.079	0.007
Female factors	299	60.0	62.75	17.32		
Male factors	56	11.3	70.49	18.18		
Mixed factors	31	6.2	63.73	14.25		
Unexplained reason	112	22.5	66.58	14.98		

Note: *QoL* indicates quality of life, *SD* indicates standard deviation

### Descriptive statistics and correlations of continuous variables

The scores of FertiQoL, age, infertility-related stress and resilience as well as the correlation coefficients between them are displayed in Table 2. The mean score of FertiQoL was 64.54 ± 16.90. Infertility-related stress was negatively correlated with fertility QoL (*P* < 0.01), whereas resilience was positively correlated with fertility QoL (*P* < 0.01). In addition, infertility-related stress was negatively correlated with resilience (*P* < 0.01).

**Table 2:** Scores and correlations of continuous variables

Variables	Mean	SD	1	2	3
1. Fertility QoL	64.54	16.90	1		
2. Age	32.19	3.83	0.048	1	
3. Infertility-related stress	145.57	24.33	−0.575**	− 0.103*	1
4. Resilience	59.53	16.18	0.535**	0.117**	−0.563**

Note: QoL indicates quality of life; SD indicates standard deviation

*indicates *P* < 0.05, ** indicates *P* < 0.01

### The moderating effect of resilience in the relationship between infertility-related stress and fertility QoL

**Fig. 1:**
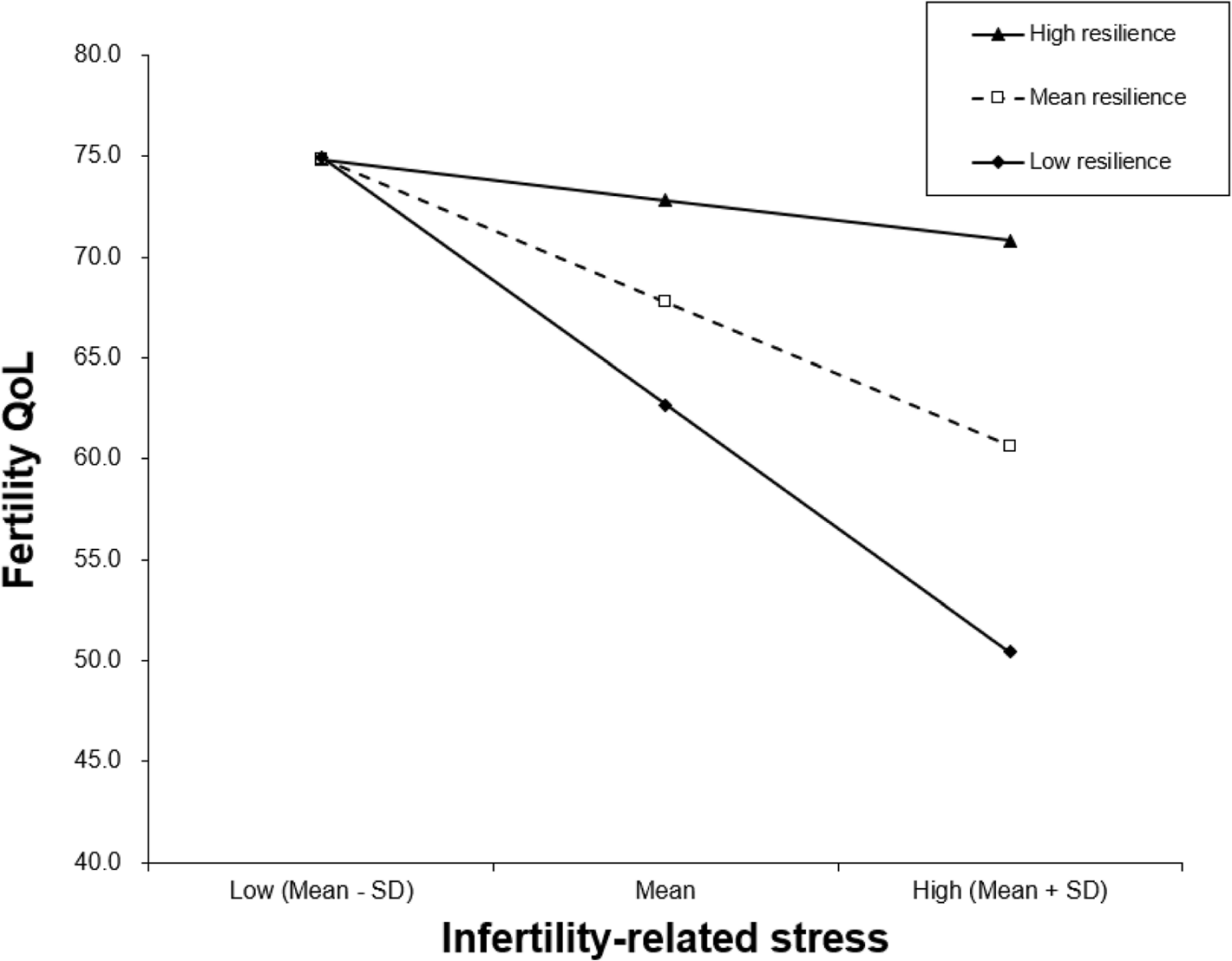
Simple slope plot of the interaction between infertility-related stress and resilience on fertility QoL. Note: The values of infertility-related stress and resilience were centered before regression analysis. Age, household monthly income and causes of infertility were adjusted. QoL indicates quality of life; low indicates 1 SD below the mean; high indicates 1 SD above the mean; SD indicates standard deviation

As shown in Table 3, hierarchical regression analysis was used to examine the moderating effect of resilience on the association of infertility-related stress with fertility QoL. In step 1, the demographic and clinical variables in the univariate analysis (*P* < 0.05) were entered as covariates, including age, household monthly income and causes of infertility. The linear combination of these control variables partially explained the variance in fertility QoL (*F* = 3.789, adjusted *R*^*2*^ = 0.027, *P* < 0.01). In step 2, infertility-related stress was found to be significantly and negatively related to fertility QoL (*B* = − 0.280, *β* = − 0.403, *P* < 0.01), while resilience was significantly and positively associated with fertility QoL (*B* = 0.316, *β* = 0.302, *P* < 0.01). Infertility-related stress and resilience exhibited significant effects on fertility QoL (*F* = 46.646, adjusted *R*^*2*^ = 0.391, Δ*R*^*2*^ = 0.363, *P* < 0.01). In step 3, the infertility-related stress × resilience interaction term was significantly and positively associated with fertility QoL (*F* = 60.528, adjusted *R*^*2*^ = 0.489, Δ*R*^*2*^ = 0.098, *P* < 0.01). Thus, resilience played a moderating role in the relationship between infertility-related stress and fertility QoL. Simple slope analysis of the interaction presented in Fig. 1, which showed that the impacts of infertility-related stress on fertility QoL were different in low (1 SD below the mean, *B* = − 0.496, *β* = − 0.714, *P* < 0.001), mean (*B* = − 0.293, *β* = − 0.422, *P* < 0.001) and high (1 SD above the mean, *B* = − 0.090, *β* = − 0.130, *P* < 0.001) levels of resilience; when resilience was higher, the effect of infertility-related stress on fertility QoL became weaker.

**Table 3:** Hierarchical regression results of fertility QoL

Variables	*B*	SE *B*	*β*	*T*	*P*-value
Step 1					
Age	0.199	0.196	0.045	1.012	0.312
Household monthly income	3.532	1.552	0.101	2.275	0.023
Causes of infertility					
Dummy_1	7.832	2.430	0.147	3.223	0.001
Dummy_2	1.722	3.159	0.025	0.545	0.586
Dummy_3	3.570	1.850	0.088	1.930	0.054
*F*	3.789				
Adjusted *R*^*2*^	0.027				
*P*-value	0.002				
Step 2					
Infertility-related stress	−0.280	0.030	−0.403	−9.477	< 0.001
Resilience	0.316	0.045	0.302	6.978	< 0.001
*F*	46.646				
Adjusted *R*^*2*^	0.391				
Δ*R*^*2*^	0.363				
*P*-value	< 0.001				
Step 3					
Infertility-related stress × resilience	0.013	0.001	0.317	9.749	< 0.001
*F*	60.528				
Adjusted *R*^*2*^	0.489				
Δ*R*^*2*^	0.098				
*P*-value	< 0.001				

Note: *QoL* indicates quality of life, *SE* indicates standard error; Dummy_1 indicates male factors vs. female factors; Dummy_2 indicates mixed factors vs. female factors; Dummy_3 indicates unexplained reason vs. female factors

## Discussion

This study evaluated the fertility QoL and its associated factors among women with infertility in China. We recruited 559 participants with a highly effective response rate of 89.1%. To the best of our knowledge, the present study was the first to explore the moderating role of resilience on the association of infertility-related stress with fertility QoL in infertile women.

We found that the mean score of FertiQoL in infertile Chinese women was 64.54 ± 16.90, which was much lower than findings in most previous studies across countries, such as Turkey (66.97 ± 14.35), the Netherlands (70.80 ± 13.90), the United States (72.30 ± 14.80), and Germany (73.00 ± 12.00) [37–40]. Despite the fact that there are affordable and advanced technologies, such as IVF-ET and easier ways to obtain access to better quality care in China, our results revealed that Chinese women with infertility still experienced poor fertility QoL. Thus, it is very imperative to determine the crucial influencing factors and targeted solutions to improve their QoL.

Among the demographic and clinical variables, only household monthly income and causes of infertility were significantly related to FertiQoL score, explaining 2.7% of the variance, which echoed the findings of previous studies [7, 9, 41]. This result indicated that the reduction of treatment costs should be considered by hospital managers to lessen the financial burden of infertile patients. Moreover, as the results showed, women with infertility caused by a female factor had the lowest FertiQoL score. Thus, the medical staff and their family members ought to be encouraged to give more care and to support to them, which may be helpful in improving their fertility QoL [42].

The results of this study showed that after controlling for the covariates, infertility-related stress and resilience were key factors affecting fertility QoL, explaining 36.3% of the total variance. Specifically, infertility-related stress was found to be significantly and negatively related to fertility QoL, which was consistent with previous studies [18, 43, 44]. Perceived stress can be considered a result of dynamic interactions between the external environment and the individual [45]. When reproductive-age women are diagnosed with infertility, this negative life event and feeling of stigma can induce heavy psychological distress. The accumulated stress can induce persistent negative emotions such as anxiety, depression and social isolation, seriously impairing patients’ QoL [28, 46]. In addition, infertility-related stress may lead to increased marital conflicts and decreased life satisfaction between wives and husbands, which can also seriously impact their quality of marital life [15, 47]. Noticeably, the mean scores of infertility-related stress of the participants in this study were found to be higher than their counterparts in other countries [17, 48, 49], which may partly explain why Chinese infertile women experienced poorer fertility QoL. Additionally, the higher level of stress can be attributed to the differences in cultures and values. In Chinese society, maternity signifies social respectability to a large extent, whereas childlessness means unfiliality (this terminology means not respecting one’s parents, elders, and ancestors). Therefore, future studies should focus more on cultural factors when identifying stressors impacting fertility QoL.

With regard to resilience, we found that it was significantly and negatively correlated with infertility-related stress and positively associated with fertility QoL, which was similar to prior studies [24, 50]. In addition, we also found that resilience moderated the association of infertility-related stress with fertility QoL, which confirmed our hypothesis. The result of the simple slope analysis showed that the higher the resilience, the weaker the effect of infertility-related stress on fertility QoL. Infertility-related stress can lead to many negative emotions and adverse physiological responses (e.g., headaches) which may overwhelm patients [51], while resilient patients are usually characterized by possessing high levels of self-esteem, self-efficacy and optimism, and they can take advantage of problem-solving skills to effectively cope with stress [20, 21]. Reasonably, infertile patients with high levels of resilience enjoyed high fertility QoL because they could recover quickly and easily from challenges in both daily life and disease conditions. This result implies that resilience as a protective factor of infertile women can reduce their perceived psychological distress, which is conducive to maintaining their physical, mental and social well-being. In addition, it highlighted the contribution of the interaction between perceived stress and resilience to fertility QoL. This means that if infertility-related stressors are hard to be effectively reduced, improving resilience may be a good way to minimize the negative impact of infertility-related stress on fertility QoL.

Based on our findings, several implications for clinical practice should be highlighted to improve fertility QoL of women with infertility. First, during the diagnosis and treatment of infertility, patients’ psychological status must be evaluated. Second, psychological counseling services should be provided to infertile couples [52]. Third, infertile patients need more social support from family members, clinicians and nurses. Finally, it is important to build resilience to improve fertility QoL in infertile women who are laden with considerable stress. As building resilience is usually considered a dynamic process, interventions such as mindfulness-based skill and cognitive-behavioral approaches could be introduced to infertile patients in the early disease stage in order to increase the protective effect of resilience on fertility QoL [53–56]. Patients could also regularly engage in a proactive personal reflective report to increase their resilience [57]. Overall, targeted intervention strategies should be conducted in future research.

Several limitations should be mentioned in the present study. First, a cross-sectional design was used in the study, and no causal conclusions could be drawn between the variables investigated. A longitudinal study should be carried out to verify our findings. Second, infertility-related stress, resilience and fertility QoL were measured using self-administered questionnaires, which could lead to possible recall bias or response bias. Third, several potential factors, such as beliefs about the importance of parenthood and personality, may affect QoL related to infertility and were not included in the study. Finally, this study was conducted in a province of the northeastern region of China. Thus, caution should be taken when extrapolating the results to infertile patients in other parts of China.

## Conclusions

Overall, women with infertility in northeastern China experienced relatively low fertility QoL. Infertility-related stress and resilience were the crucial factors associated with fertility QoL, and resilience moderated the association of infertility-related stress with fertility QoL. Thus, in clinical practice, more attention should be paid to psychological stress, and more social support should be provided to Chinese women with infertility. More importantly, adequate resilience interventions such as mindfulness-based skills should be introduced to improve their fertility QoL.

## Abbreviations

CD-RISC: Connor-Davidson Resilience Scale
FertiQoL: Fertility quality of life
FPI: Fertility Problem Inventory
QoL: Quality of life
SD: Standard deviation
SE: Standard error
WHO: World Health Organization
